# How people with disabilities influence crowd dynamics of pedestrian movement through bottlenecks

**DOI:** 10.1038/s41598-022-18142-7

**Published:** 2022-08-22

**Authors:** Paul Geoerg, Jette Schumann, Maik Boltes, Max Kinateder

**Affiliations:** 1 Otto-von-Guericke University, Magdeburg, Germany; 2 Forschungszentrum Jülich GmbH, Jülich, Germany; 3 National Research Council Canada, Ottawa, Canada; 4 EvacTrain GmbH, Magdeburg, Germany

**Keywords:** Civil engineering, Fluid dynamics

## Abstract

Despite considerable research efforts, most controlled empirical studies on crowd movement usually rely on homogeneous crowds, i.e., research participants are typically young adults without disabilities. Consequently, relatively little is known about pedestrian movement in more diverse and heterogeneous crowd conditions, e.g., when persons with reduced mobility are present. This gap may be particularly relevant at bottlenecks, along the path of a moving crowd, that limit the capacity of pedestrian flow. Here, we present results from 12 studies in which participants (total *N* = 252) with and without visible disabilities moved together in a crowd. In each study, groups of participants walked together in a hallway with a bottleneck at the end. The point of speed adoption, distances between neighbours, and behavioural activities were analysed. We found (1) that participants with disabilities reduced their speed further away from the bottleneck than participants without disabilities; (2) participants without disabilities stayed closer to neighbors with disabilities than to neighbors without disabilities; and (3) participants interacted and communicated with each other to organise in front of the bottleneck. These results underline the importance of studying representative and heterogeneous samples in crowd dynamics. We also argue that more interdisciplinary research is needed to better understand the dynamics of interactions between neighbors in a crowd. A more nuanced understanding of pedestrian dynamics holds the promise of improving the validity of simulation tools such as movement and evacuation models.

## Introduction

For the approximately 15.6 % of the world’s population who are living with disabilities^1^, safely navigating within crowds can pose unique challenges. The quantity and quality of controlled laboratory and field studies on pedestrian movement dynamics has increased dramatically in the last decades^2–5^, so that it is increasingly possible to generate performance benchmarks related to occupant movement^6^. While these studies have improved our understanding of pedestrian dynamics, individual differences in mobility have only rarely been considered^7^. More generally, criticism has been raised pointing to the lack of environmental and contextual realism in study design as well as the disregard of the influence that interactions between neighbors in a crowd have on global patterns of motion^8^.

In recent years, several studies have expanded the classical experimental settings to address psychological questions in pedestrian movement dynamics. Adrian et al.^9^ present a physical and social psychological analysis of pedestrians moving through a bottleneck. The authors found that behaviours such as pushing were perceived as unfair by the participants, but were used as a possible strategy to get through the bottleneck more quickly. These findings are in line with a previous work of the same group. For example, Sieben et al.^10^ identified a relationship between the geometry of the environment, the density of pedestrians, and the pedestrians’ motivation (i.e., their desire to be among the first to move through the bottleneck) as well as their behavioural strategies for faster access (e.g. pushing).

Another line of research explores the role of collective identity within a crowd and its effects on pro or antisocial behaviour during mass evacuation situations. Drury et al.^11^ found that individual support offers depended on perceived threat. In addition, the reaction of the study participants depended on whether their personal or collective identities were salient. The authors conclude that collective identity is a key predictor of pro and antisocial actions (e.g. pushing, cooperation) in crowds.

Improved tracking methods and emerging technologies, e.g. virtual or augmented reality methods^12^ offer more and more behavioural insights into crowd motion and individual behaviour^13^. For example, Rio et al.^14^ studied the local interactions (e.g., alignment in movement speed and heading direction) in pedestrian crowds that produce a global (i.e., crowd level) pattern of motions^15^. They present a model of speed-alignment between pedestrians and their neighbours and describe a circular symmetric local neighbourhood around a pedestrian. In this neighbourhood, pedestrians are coupled uni-directional to neighbours within $\pm {90}^{\circ }$ of their current heading. This raises the question of whether the analysis of the area and neighbourhood of interaction can also provide valuable results suitable for extending existing models to include aspects of interaction and individual behaviour.

## Current research

Pedestrians headway at the entrance of bottlenecks is of particular interest because the time-lapse between two consecutive participants can be derived as flow *J*^16^. A linear relation between flow rate and bottleneck width for different passage width *w* and populations is reported^17–19^.

The investigation of *one-dimensional flows* (i.e., pedestrians walking behind each other in one direction) allows to reduce the complexity and to focus on longitudinal interactions between pedestrians. In single-file motion experiments, Jelic et al.^20^ found different linear relationships with different slopes between heading and speed, especially if larger density ranges were covered. Interestingly, the correlation between heading and speed was stronger in distances closer than 1.1 m to the person walking in front, while larger distances lead to a decrease in the correlation. These findings were also confirmed for populations of different ages^21,22^ or for people carry luggage^23^. Ma et al.^24^ investigated the relation between step length (or step duration) and heading. Similar to findings on speed-dependency, they found a linear relation between step length and heading. Again, this relation was dependent on heading, with a stronger correlation in distances smaller than 1.2 m.

Furthermore, *interpersonal distance* in crowds has been investigated. Bandini et al.^25^ focused on proxemics and pedestrians space in dynamic situations. They observed an ellipsoid spatial buffer around moving pedestrians. In a study on obstacle avoidance while moving in a crowd, Alhawsawi et al.^26^ found an impact of speed on lateral movement which correlates with increasing speed is reported. In a study on uni- and bidirectional flow, Cao et al.^22^ showed that, when pedestrians – who were moving in different directions – tended to increase the individual proximity to maintain their unimpeded movement speed.

Understanding the *(inter-)relations between neighbours* in a crowd can be used to asses the social impact on movement parameters (e.g., speed or maintained proximity). Studies on social interaction in crowds and its influence on interpersonal distances are rare. Templeton et al.^27^ report a slower speed and a closer proximity in a psychological crowd compared to physical crowds. The preferred interpersonal distances, understood as a maintained distance to neighbours or walls that support regulation of intimacy, was compared across cultures in a meta-analysis by Sorokowska et al.^28^ based on data collected via questionnaires. The authors found an influence of age and gender on maintained proximity, as well as cultural differences.

To understand the influence of individual behaviour on a crowd as a whole, a refined, conceptual understanding of the effect of individual behaviour in the temporal-spatial context of the movement process is required. Depending on the unique research goals, data on pedestrian dynamics is generated in varying ways. For the abstract representation of interactions between individuals, graphic notation systems are used in other disciplines (e.g. in dance and theatre sciences, fire cause or crime scene investigation)^29^. Presented a spatio-temporal mapping approach on evacuee response using a notation system to document evacuation procedures and interpersonal interaction graphically. Similarly^30^, addresses the behavioural mapping in geolocation in urban space. Both methods provide a temporal and spatial representation of individual events (e.g. behavioural actions) and their standardised notation.

This work continues on the investigation of proxemics and accompanying social behaviour. For this purpose, data of laboratory studies on the movement of a heterogeneous crowd through a bottleneck are analysed.

## Methods

The analyses presented here are based on empirical studies of pedestrian movement (see below and^31–34^). In multiple trials, crowds of pedestrians with and without disabilities moved through a simulated hallway before passing a bottleneck.

### Independent variables

The following independent variables were manipulated.

1. *Crowd heterogeneity* refers to the composition of the crowd in a given trial (three levels).
  - The *wheelchair group* (whe): A group of participants using a wheelchair (whe) moving with participants without disabilities (PWoDs).
  - The *mixed group* (mix): A group of participants with disabilities (PWD) and assistive devices as a self-operated manual or electric wheelchair, white cane, or personal assistance moving with PWoDs.
  - A *reference group* (ref) of PWoDs.
2. *Bottleneck width* refers to the width of the bottleneck through which the crowd moved (two levels).
  - 0.9 m
  - 1.2 m

Table 1 gives an overview of the experimental design as well as the number of participants in each condition. Each crowd heterogeneity condition was repeated twice per bottleneck width with a different *subset* of participants (labeled A1, A2, B1 ... F2). Each subset of participants repeated each condition twice, yielding a total of 24 trials.

**Table 1:** Overview of experimental conditions, crowd composition, and number of frames in each trial.

Whe	Subset	A1	A2	B1	B2
	Bottleneck width	0.9 m		1.2 m	
	First steady state frame	600	600	750	500
	Last steady state frame	1600	1800	1040	1100

Mix	Subset	C1	C2	D1	D2
	Bottleneck width	0.9 m		1.2 m	
	First steady state frame	400	700	550	400
	Last steady state frame	890	1500	780	810

Ref	Subset	E1	E2	F1	F2
	Bottleneck width	0.9 m		1.2 m	
	First steady state frame	420	500	600	480
	Last steady state frame	800	705	750	650

Note that some participants rested between trials. On these occasions, volunteers who supported the experimental procedure occasionally completed those trials instead.

### Study configuration

**Figure 1:**
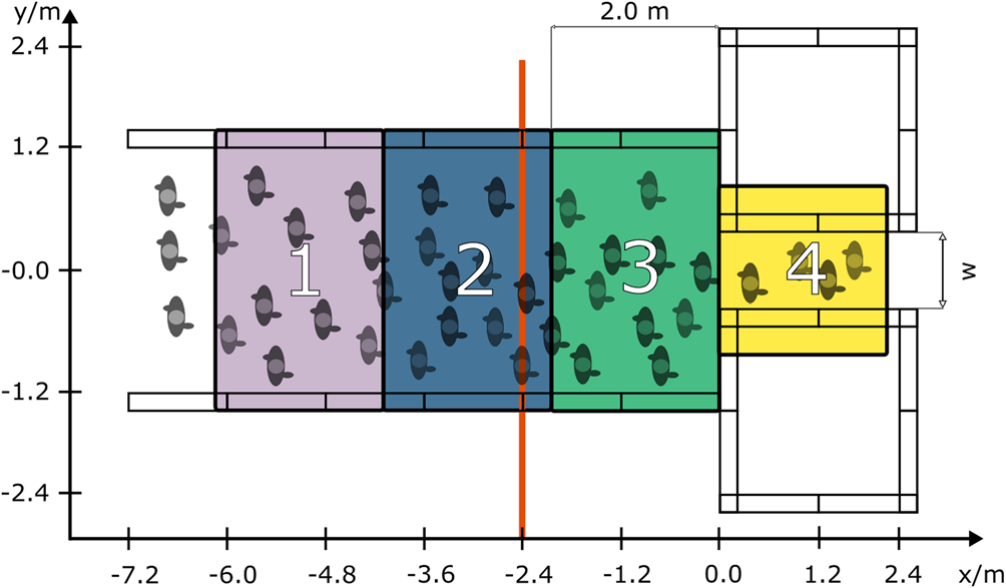
Study configuration. The passage width *w* varied between 0.9 m and 1.2 m. Measurement areas regarding the behavioural action analysis are presented as coloured boxes. The line for the spatio-temporal analysis (Fig. 8) is located 2.4 m in front of the bottleneck (vertical orange line).

A simplified geometric representation of a bottleneck situation (Fig. 1) characterised by different passage widths *w* of 0.9 m and 1.2 m was used. The length of the bottleneck was set as constant to 2.4 m.

Boundaries were built from wooden three-layer panels with a height of 2.0 m to generate the impression of a structural boundary for the participants. A waiting and starting area of $\approx {30} \text {m}^{2}$ was located at a distance to the bottleneck of 12 m with an initial density of about ${3.0} \text {m}^{-2}$.

Favoring the distance between the waiting area and the bottleneck, a heterogeneous density pattern appeared at the entrance to the bottleneck. Participants with high individual speed were at the head of the crowd, while slower participants were overtaken. This led not only to a spatio-temporally heterogeneous distribution, but also to a slightly lower density at the bottleneck entrance. This process was intentionally tolerated for the following reasons: First, the context of the present studies is movement under everyday conditions. Very high densities ($\ge$ 5 m$^{-2}$) that may occur during emergency conditions, are of course very interesting especially for a fundamental diagram analysis or for the analysis of congestion but go beyond the scope of the present paper. Second, the study was purposely designed to allow overtaking or priority maneuvers. This inevitably decreases the frequency of high densities. Third, high density conditions were excluded in the planning for ethical reasons. Since vulnerable populations with physical, mental, or age-related impairments participated in the studies, the experimenters agreed on a maximum density of 5 m$^{-2}$ in the study area.

### Study procedure

Data were collected under controlled conditions over the course of two days in June 2017. In addition to the data reported here, data for several other research questions were recorded and are beyond the scope of the present analysis (see also^32,34^.

Groups were instructed orally to move directly and briskly through the bottleneck, but without pushing.

### Participants

Overall, 252 volunteers with and without disabilities participated in the studies. The body height of all participants ranged between 1.05 m and 2.04 m with a mean of ${1.74}{} \pm {0.1} m$. Body height was measured prior to data collection and defined as the distance from floor to the top of a participant’s head (either standing or sitting in a wheelchair). Table  2 shows the demographic information for each of the three crowd heterogeneity groups.

**Table 2:** Demographic information.

Crowd heterogeneity	Disability	*n*	Age (mean $\pm {}$ sd)	Gender m/f
Whe	PWoD	84	${35.9} \pm {16.3}$	40/44
Whe	PWD	7	${47.6} \pm {7.0}$	2/5
Mix	PWoD	73	${31.5} \pm {15.1}$	34/39
Mix	PWD	5	${42.2} \pm {13.0}$	1/4
Ref	PWoD	71	${32.1} \pm {15.5}$	35/36

Since PWDs varied greatly in their mobility, individual mobility profiles of each PWD are shown in more detail in Table 3.

**Table 3:** Mobility profiles of PWDs including the length $l_{whe}$ and the width $w_{whe}$ of the wheelchair (cf. Supplementary Data 3 for more details on the description of mobility characteristics).

Participant	Crowd heterogeneity	Assistive device	Gender	Age	Details
1	Whe	Manually operated wheelchair	f	38	$l_{whe}= {1.40}m$, $w_{whe} = {0.60}m$ with personal assistance
2	Whe	Manually operated wheelchair	m	56	$l_{whe}= {1.16}m$, $w_{whe} = {0.74}m$ operates wheelchair single-handedly
3	Whe	Electrically operated wheelchair	f	53	$l_{whe}= {1.23}m$, $w_{whe} = {0.64}m$
4	Whe	Electrically operated wheelchair	f	47	$l_{whe}= {1.10}m$, $w_{whe} = {0.77}m$, obese participant
5	Whe	Manually operated wheelchair	f	40	$l_{whe}= {0.92}m$, $w_{whe} = {0.66}m$
6	Whe	Manually operated wheelchair	f	43	$l_{whe}= {0.90}m$, $w_{whe} = {0.60}m$
7	Whe	Manually operated wheelchair	m	56	$l_{whe}= {0.76}m$, $w_{whe} = {0.66}m$
8	Mix	Not assisted	f	45	legally blind
9	Mix	Not assisted	f	61	severe auditory impairment
10	Mix	Manually operated wheelchair	m	32	$l_{whe}= {1.10}m$, $w_{whe} = {0.80}m$
11	Mix	Assisted by others	f	49	difficulties in orientation and dependent on hand-guidance by others
12	Mix	White cane and assisted others	f	24	orientation by hand and uses of arms for orientation

For more details regarding the recruiting and participant selection process, please see^35^.

### Research ethics

The study protocols followed the principles of the Declaration of Helsinki^36^. Participation provided their informed consent.Consent to participate could be withdrawn at any time and for any reason (cf. SupplSupplementary Data 4). All participants were compensated for their time. Only de-identified data were used for the studies and the methodological design, data storage process and the access authorisation for data were approved by the ethics committee of the Bergische Universität Wuppertal, Germany.

### Data analysis

#### Data extraction

Trajectories for each participant were generated from raw image data. The setup was captured by nine high-definition video cameras with a framerate of 25 s$^{-1}$. Trajectories were extracted from the raw image data with *PeTrack*^37^, using the following procedure: Each participant wore a cap with a specific colour to track their position at higher densities. The colour of the caps was chosen in based on a participant’s body height to reduce the error coming from the perspective distortion. The centre of the pixel areas corresponding to a unique colour code represents the position of a participant in a given frame. Assuming that a coherent area of pixels in the middle of a cap can be used as a proxy for a pedestrian body centre, the resulting trajectories represent positions of the head projected along x and y-coordinates on the ground. Trajectories were checked and corrected manually, if necessary. For more details on the procedure, see Boltes 2013^37^.

#### Dependent variables

In order to analyse the relation between neighbours, speed *v* and interpersonal distance *d* were retrieved from the trajectory data. Speed was calculated using the Voronoi method, and distances between neighbours were approximated as Delaunay edges. In addition, behavioural activity was quantified by means of qualitative video analysis and is described in detail in “Observation of behavioural activity” section.

#### Measurement of speed and distances between neighbours

**Figure 2:**
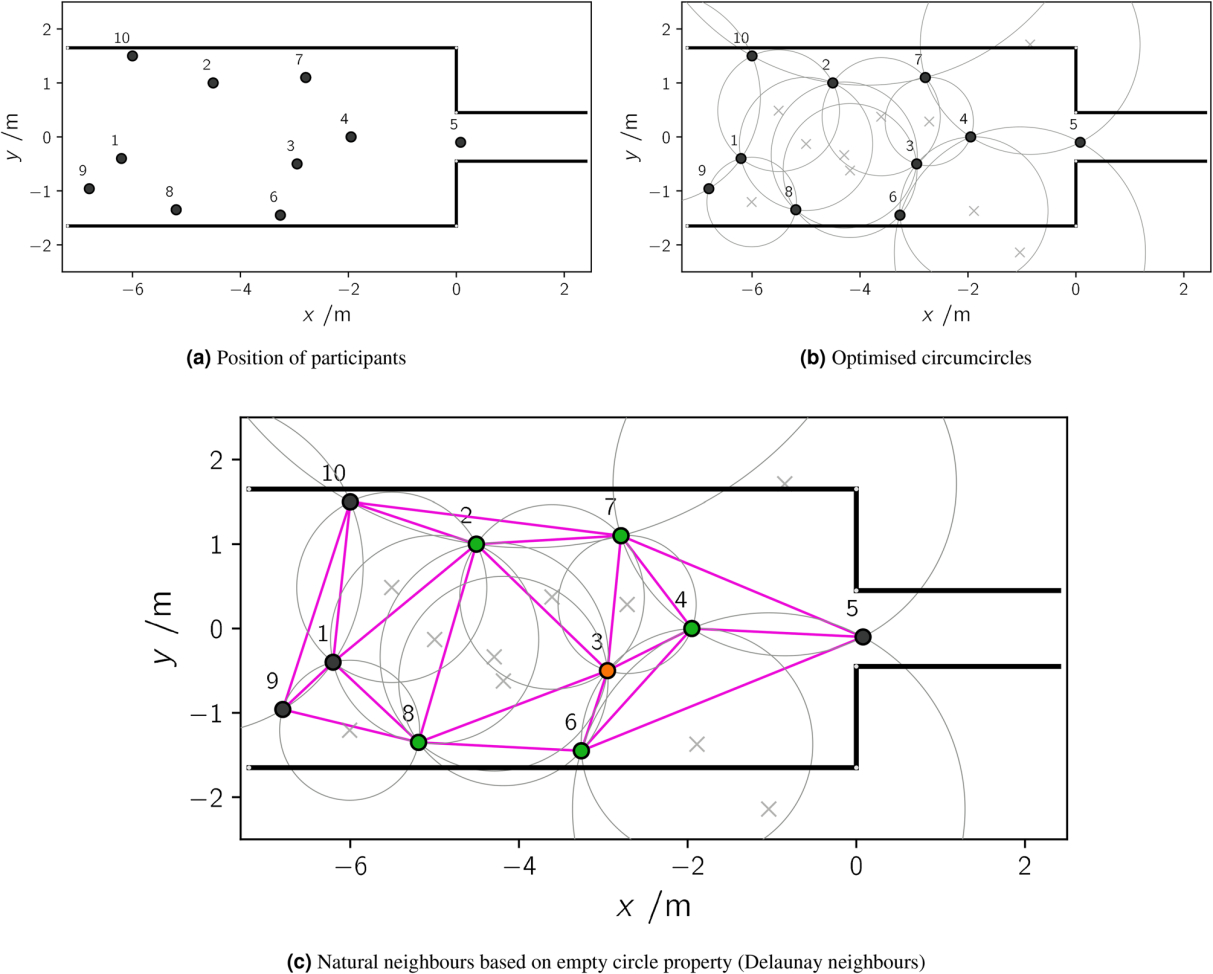
Principle of a Delaunay triangulation presented for a set of ten trajectory points. (**a**) Ten trajectory points in a bottleneck setting; (**b**) calculation of a unique circle that passes through each trajectory point and has no other point in his interior; (**c**) triangulation of the point set satisfying the empty circle property (Delaunay triangulation); Neighbours (green) of a participant of interest (orange) are defined as trajectory points connected by Delaunay edges (pink lines).

The interpersonal distance was determined using the Delaunay triangulation and is based on the assumption that a participant’s position in space and time $P(t) = (x,y)$ can be represented as a set of points in a two-dimensional metric space (Fig. 2a). If a participant is surrounded by at least two other participants, then these are defined as neighbours, if their three position points are defined as connected to a triangle, as long as the interior of a unique circumcircle does not contain another point (Fig. 2b). This is referred to as the empty circle property^38^, which is summarised and discussed in detail by Okabe et al.^39^. For each edge in a Delaunay triangulation there is an empty circle property through the end points of the edge (Fig. 2c). The points on the circumcircle are called *natural neighbours*^39^ and the smallest interior angle for all triangles inside this mesh is maximised. In addition, the Delaunay edge is restricted by walls, obstacles, and generally also by a cutoff radius $r_{cut}$ (here set to 2.0 m) that represents the participant’s maximum circle of influence.

The definition of a participant’s neighbourhood in a Delaunay triangulation is hypothetically exemplified, presented in Fig. 2. Neighbours of a given participant (persID) 3 (orange)—here defined as points with shared edges of triangles (natural neighbours)—are coloured in green. Therefore, the individual distances *d* between neighbours for the purpose of this paper are the Delaunay edges. Participants coloured in black (persID $\in [1,5,9,10]$) are not neighbours (Fig. 2c). Circumcircles and circumcenters of the resulting triangles are indicated in light grey.

A particular characteristic of Delaunay triangulation is that its edges are perpendicular to the boundaries of Voronoi cells $A_{i}(t)$, which crosses the edges in their middle. A Voronoi cell assigns an individually required space to each person (similar to the individually covered space). All points in the Euclidean plane of this Voronoi cell are closer to a participant *i* than to all other participants (^40–42^). Thus, speed inside a Voronoi cell $A\_i$ is set to the individual speed $v_{i}$ for a participant *i* at position (*x*, *y*) and his Voronoi cell $A_{i}$ at time *t*:

1 $$\begin{aligned} v_{i} (x,y,t)&= {\left\{ \begin{array}{ll} v_{i} (t) &{} \text {, } \forall (x,y) \in A_{i} (t) \\ 0 &{} \text {, } \text {other} \end{array}\right. } \end{aligned}$$

With speeds (1) related to individual positions, the average Voronoi speed $v_{v}$ is obtained at time step *t* inside the measuring area $\Delta x \cdot \Delta y$:

2 $$\begin{aligned} v_{v} (t, \Delta x, \Delta y)&= \frac{1}{\Delta x \cdot \Delta y} \iint v_{i} (x,y,t) \mathrm {d}x \mathrm {d}y \end{aligned}$$

The time that passes between two participants entering the bottleneck is referred to as a ‘time gap’ $\Delta t_{i}$. The flow rate *J* based on individual time gaps $\Delta t_{i}$ between *N* participants is similar to the number of participants crossing the fixed location at $x = 0$^43^. Therefore, the time gaps $\Delta t_{i} = t_{i+1} - t_{i}$ are calculated between the consecutive participants *i* and $i+1$. The sum of individual time gaps is directly related to the flow rate $J= \frac{1}{\Delta t_{i}}$.

#### Observation of behavioural activity

In order to obtain an exploratory insight into the influence of the social interaction within the crowd, a standardised procedure to quantify the occurrences of behavioural actions was developed. For this purpose, the study area was organised into four measurement areas (Fig. 1), in which behavioural actions were counted on the basis of the video recordings.

Twelve observable items were identified in a proof of concept, such as turning around and gestures (cf. Supplementary Data 2 and Supplementary Data 3). In addition to actions of observable behaviour, this list also includes a method to describe visual attributes of a person, including disabilities based on their phenotype (such as the use of a wheelchair). The procedure to quantify behavioural actions was organised as follows:

1. *Observational overview*: For an overview of which items from the preliminary study are applicable to the studies selected for analysis, the first trial for each subset setting with a bottleneck width of 0.9 m was selected for analysis. The observed behaviours were defined and documented.
2. *Revision of definitions*: The behavioural definitions were revised and a collection of generic categories such as ‘gestures’ or ‘assisted movement’ was created in an iterative process.
3. *Operationalisation*: The behavioural actions were finalized and a specification of a coded description was determined (e.g. participant turning the head or the upper body away from the main direction of movement by at least 30°  for more than 1 s). Standard examples were applied and summarised in a coding manual (cf. Table 4 for an example).
4. *Coding 1*: Footage was coded in each measurement area using the coding manual. If needed, minor revisions of the coding manual were made.
5. *Coding 2*: The footage was coded twice by the same researcher to ensure reliable results.

**Table 4:** Excerpt of the coding manual.

Category	Subcategory	Description
Gesture$^{a}$	one arm	A gesture made with one arm or hand and addressed to another person.
	two arms	A gesture made with both arms or hands and addressed to another person.

$^{a}$ Excluded are: Leaning on another person or wall, running fingers through the hair, covering the mouth with hands while coughing, putting hands in pockets, and folding arms.

Please note that the original coding manual contains standard examples from the original video. These examples have been removed for publication for data protection reasons, despite the consent of the participants.

## Results

**Figure 3:**
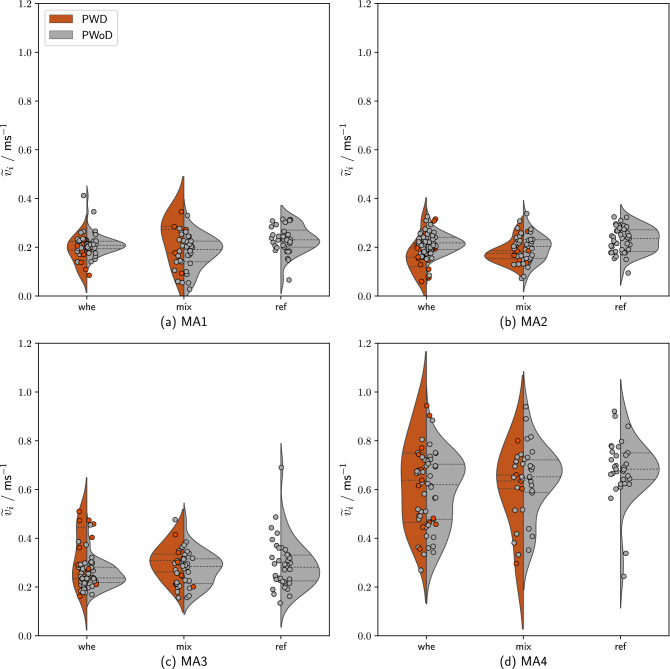
Speed as a function of *crowd heterogeneity* and *MA*. Note: the analysis was carried out in a stationary time interval (cf. Table 1). Consequently, only data points from participants who were in the measurement areas at the defined time interval were considered. The horizontal lines in the violin chart indicate the median as well as 25% and 75% quantiles. Please note that the position of the data points on the x-axis is of no relevance and only avoids that they overlap each other.

The results section begins by comparing speed and interpersonal distance between the three experimental groups across the four measurement areas (MAs). Next, we analysed the flow rate at the bottleneck. Finally, in order to explore potential explanations for the emerging patterns in crowd dynamics, we provide an analysis of behavioral activities (e.g., hand gestures) that were observed during the trials. Overall, movement in our study was characterised by fluctuations in speed (Fig. 3) and interpersonal distance (Fig. 5).

To describe movement dynamics on an aggregated level, movement speeds were summarized as simple moving averages for each individual participant (Fig. 3) and interpersonal distance (Fig. 5) were averaged across participants and the four MAs. For each dependent measure, we first report on differences in bottleneck width, location (i.e., the four MAs), crowd heterogeneity, and finally explore differences between PWDs and PWoDs in the *whe* and *mix* groups.

### Statistical analysis

The following approach was used for statistical significance testing. We assumed that the data in different conditions are independent of each other. We then tested whether the outcome variables (e.g., speed) were normally distributed (using Shapiro-Wilk tests^44^). In most cases, the dependent variables were *not* normally distributed, and we used rank-based nonparametric tests (Kruskal-Wallis test^45^) to determine if there were statistically significant differences between groups on the dependent variable.

The significance level was set at $\alpha$ = 0.05 for all statistical analysis. Where necessary, significance cut-offs were adjusted for multiple testing using the Bonferroni-Holm method. All statistical analysis was carried out using^46^. Results of statistical tests are reported following guidelines of the American Psychological Association^47^.

### Speed

Figure 3 shows movement speed by *crowd heterogeneity* in the four MAs. Visual inspection suggests that the distribution and central tendency of speed differs by MA and population: Descriptively, participants were moving faster as they approached the bottleneck and especially after passing the bottleneck (Fig. 3). However, note that the distribution of speed (especially for PWDs) became wider (see violin plots). Since average speed was not normally distributed in any of the MAs or overall (all Shapiro-Wilk tests: *p* < .01), non-parametric Kruskal-Wallis tests were used to test for effects of *crowd heterogeneity* and *MA* on average movement speed.

#### Effect of bottleneck width

We observed significant differences between the two bottleneck widths in speed, $\chi ^2 (1) = 46.15$, *p* < .001. When broken down by MA, we found that participants consistently moved faster in all MAs (up to 0.05 m$^{-1}$ on average; all $p < .001$), except for MA4 (no significant differences).

#### Effect of location

Speed varied significantly between the four MAs, $\chi ^2 (3) = 671.63$, $p < .001$. Follow-up Wilcoxon rank sum tests showed no significant differences between MA1 ($\widetilde{v_{i}} = {0.21} \text {m}^{-1}$) and MA2 ($\widetilde{v_{i}} = {0.22} \text {m}^{-1}$), ($p = .46$), but between all other MAs (all $p < .01$), with higher speeds in MA3 ($\widetilde{v_{i}} = {0.29} \text {m}^{-1}$) and even higher speeds in MA4 ($\widetilde{v_{i}} = {0.67} \text {m}^{-1}$).

#### Effect of crowd heterogeneity

We then compared median speed between the three *crowd heterogeneity* conditions. Across all MAs, the *whe* group (median = ${0.276} \text {m}^{-1}$), moved significantly slower than the *ref* group ($\widetilde{v_{i}} = {0.291} \text {m}^{-1}$;*p* < .05) but not the *mix* group ($\widetilde{v_{i}} = {0.245} \text {m}^{-1}$;*p* = .23), $\chi ^2$ (2) = 8.05, *p* < .05. There were no significant differences between the *mix* and the *ref* group, *p* = .18.

When testing for effects of *crowd heterogeneity* on speed within each of the four MAs, we found significant effects of *crowd heterogeneity* in MA1, $\chi ^2$ (2) = 10.45, *p* < .01, and MA2, $\chi ^2$ (2) = 14.26, *p* < .001, but not in MA3, $\chi ^2$ (2) = 4.86, *p* = .08, and MA4, $\chi ^2$ (2) = 0.33, *p* = .84. Follow-up comparisons, showed that the *whe* was moving significantly slower than the *ref* group in MA1, *p* < .05. In MA2, both the *whe* and the *mix* group were moving significantly slower than the *ref* group, all *p* < .01.

#### Differences between PWDs and PWoDs

Finally, we explored whether PWDs moved at different speeds than PWoDs. In the *mix* group, there were no significant differences in neither of the four MAs or overall. However, there were some differences in the *whe* group; In MA2, PWDs ($\widetilde{v_{i}} = {0.17} \text {m}^{-1}$) moved significantly slower than PWoDs ($\widetilde{v_{i}} = {0.21} \text {m}^{-1}$, *p* < .01). The inverse pattern could be observed in MA3, where PWDs ($\widetilde{v_{i}} = {0.31} \text {m}^{-1}$) moved significantly faster than PWoDs ($\widetilde{v_{i}} = {0.26} \text {m}^{-1}$, *p* < .05).

### Distances between neighbours

Average distance between neighbours within the MAs provides insight into how much space people are willing and able to give each other. Surprisingly, it is not only the area immediately in front of the bottleneck that can be of particular interest, but also MAs further upstream (as introduced in Fig. 1). The distances between neighbours varied as a function of the distance to the bottleneck and *crowd heterogeneity* (cf. Figd. 4 and  5).

**Figure 4:**
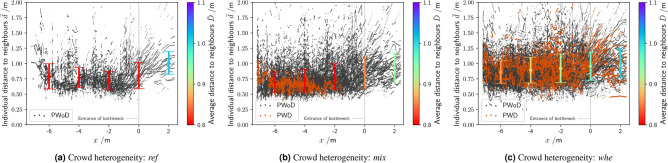
Individual $\bar{d}$ and average distance $\bar{D}$ between neighbours situation with a passage width of 0.9 m. (**a**) presents data for PWoD only in the *ref* group, (**b**) presents data for the *mix* group, and (**c**) presents data in the (*whe*) group.

Since average distance between neighbors was not normally distributed in MA1 and MA3 (all Shapiro-Wilk test: *p* < .01), Kruskal-Wallis tests were used to test for effects of *Crowd heterogeneity* on average distance between neighbors.

#### Effect of location

There were no significant differences in distances between the four MAs, $\chi ^2 (3) = 3.23, p = .356$.

#### Effect of Crowd heterogeneity

We then compared median distance between the three *crowd heterogeneity* conditions. Across all MAs, we found an effect of *crowd heterogeneity*, $\chi ^2 (2) = 140.24, p < .001$, with distances being largest in the *whe* = 0.89 group, followed by *ref* = 0.86, and *mix* = 0.80 (all $p < .001$).

When testing for effects of *crowd heterogeneity* on distance within each of the four MAs, we found significant effects of *crowd heterogeneity* in MA1, $\chi ^2 (2) = 52.29, p < .001$, MA2, $\chi ^2 (2) = 39.15, p < .001$, MA3, $\chi ^2 (2) = 30.93, p < .001$, and MA4, $\chi ^2 (2) = 20.61, p < .001$. In each MA, we found that distances were largest in the *whe* group, followed by the *ref* and *mix* group. Follow-up comparisons confirmed this pattern, with significant differences for all but differences between *ref* and *mix* groups in MA3 and MA4 (*p* < 0.01). In addition, no significant difference was found between the *ref* and *whe* group in MA4.

#### Differences between PWDs and PWoDs

For the most part, we did not find significant differences in median distance between PWDs and PWoDs, with one exception. In the *mix* group, however, PWDs kept a significantly larger distance (median = 0.85 m) from other people than PWoDs ($\widetilde{D_{i}} = {0.79}m$), $\chi ^2$ (1) = 5.09, *p* < .05. Note, however, that the difference between the median average distances is around 0.05 m.

**Figure 5:**
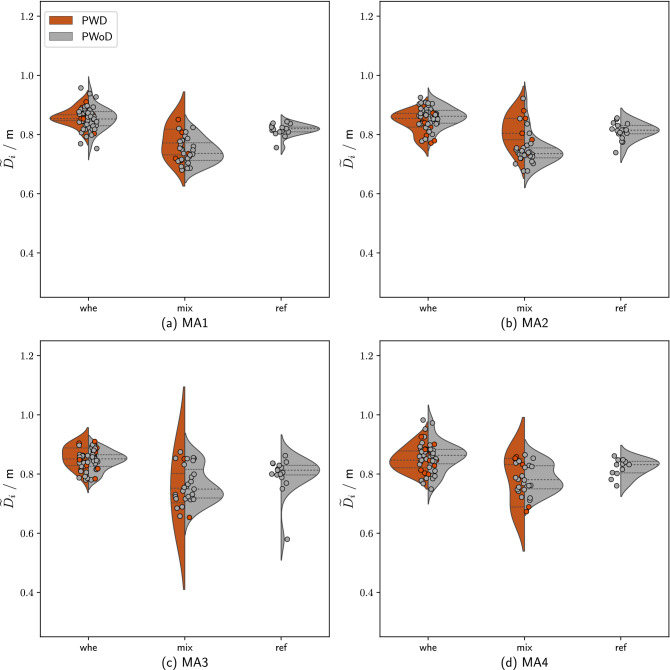
Median distance to neighbours $\widetilde{D}_{i}$ in the four MAs as a function of *crowd heterogeneity*. Notes: the analysis was carried out in a stationary time interval (cf. Table 1). Consequently, only data points of persons who were in the MAs at the defined time interval were considered.

### Flow rate

**Figure 6:**
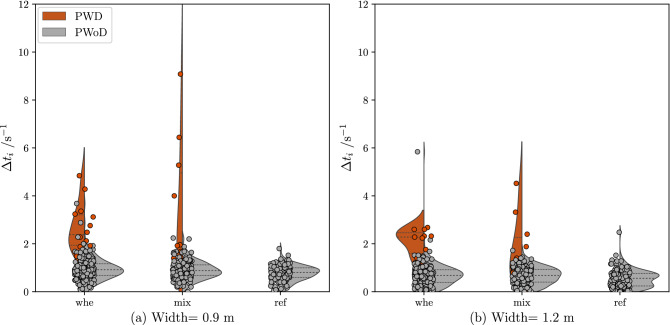
Individual time gap $\Delta t_{i}$ as a function of *crowd heterogeneity* and bottleneck *width*.

In addition to speed and interpersonal distance, we also measured the flow at the bottleneck (i.e., at the line between MA3 and MA4; Table 5). We observed lower flow rates in the two heterogeneous groups compared to the *ref* group (Table 5). We then analysed the time gap data in more detail. Figure 6 shows time gaps as a function of *crowd heterogeneity* and for the two bottleneck *widths*.

Interestingly, the time gap data were not normally distributed (all Shapiro-Wilk test: $p < .01$), except for the time gaps in the *ref* group in the more narrow bottleneck (0.9 m). Kruskal-Wallis tests were used to test for effects of *crowd heterogeneity* and *width* on time gaps.

#### Effect of width

Time gaps were significantly smaller in the wider bottleneck, $\chi ^2 (2) = 81.78, p < .001$, resulting in higher flow rates (Table 5).

#### Effect of crowd heterogeneity

We then compared time gaps in the three *crowd heterogeneity* conditions. Not surprisingly, we found an effect of *crowd heterogeneity* on the length of time gaps, $\chi ^2 (2) = 30.55, p < .001$, with time gaps being significantly shorter in the *ref* group ($\widetilde{\Delta t_{i}} = {0.38} \text {s}$ ) than in the *whe* ($\widetilde{\Delta t_{i}} = {0.84} \text {s}$) and the *mix* ($\widetilde{\Delta t_{i}} = {0.80} \text {s}$) groups (all $p < .001$). There were no significant differences between the two heterogeneous groups.

**Table 5:** Flow depending on crowd heterogeneity.

Crowd heterogeneity	Flow *J* s$^{-1}$	
	w = 0.9 m	w = 1.2 m
whe	0.91 ± 0.03	1.28 ± 0.06
mix	0.95 ± 0.05	1.39 ± 0.11
ref	1.30 ± 0.00	1.85 ± 0.08

### Behavioural activity

**Figure 7:**
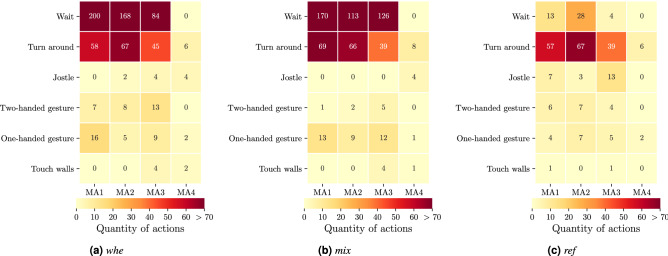
Frequency of observable behavioural actions across the four MAs and in the three experimental groups.

Six measurable behavioural actions were quantified; waiting, turning around, jostling, one-handed gestures, two-handed gestures, and touching walls. Figure 7 aggregates the actions for the three experimental groups across the MAs. ‘Waiting’ and ‘Turning around’ were the most commonly observed behaviours in all study configurations and populations, regardless of crowd heterogeneity. Participants were much more likely to stop and ‘wait’ in the *mix* and *whe* groups compared to the *ref* group. While in the two groups of higher crowd heterogeneity, participants ‘waited’ hundreds of times, this behavior was only observed occasionally in the homogeneous group. The frequency also decreased the closer participants got to the bottleneck entrance (*whe*), seemed to be constant from 4 m before the bottleneck (*mix*), or decreased noticeably 2 m before the bottleneck entrance (*ref*).

The number of one-handed and two-handed gestures differed between *crowd heterogeneity* conditions. While in the *ref* group, gestures were only sporadically observed (these actions were almost exclusively performed in the context of conversations), this means of communication was used more frequently in heterogeneously composed groups. Again, the number of gestures decreases after the bottleneck and increased the further away participants were from the bottleneck.

Participants touched the walls only sporadically in all studies. When they did so, this was most frequently immediately in front of the entrance to the bottleneck.

Somewhat surprisingly, we occasionally observed ‘jostling’, particularly in the *ref* group. These were almost without exception, participants walking at the end of the crowd and towards the end of data collection. In addition, almost always the same participants were involved in these actions.

## Discussion

Here, we reported on 12 trials of large-scale movement studies under laboratory conditions. The studies tested the effects of *crowd heterogeneity* and *bottleneck width* on speed and interpersonal distance, as well as overall flow rates at the bottleneck. We then explored measurable behavioral activities that could potentially explain differences in the main dependent variables.

The most consistent result observed across studies was that participants with disabilities (PWDs) differed in speed from participants without disabilities (PWoDs). These effects then explain differences in flow rates on a more aggregate level. However, the overall crowd dynamics were not only influenced by the crowd composition, but also by the boundaries of the environment. For instance, speed and interpersonal distance were largely independent of crowd heterogeneity in MA1 and MA4. In these sections, the crowd dynamics were largely driven by the geometry of the experimental setup. Another consistent finding across most measures was that increased heterogeneity at times lead to dramatic changes in data distribution. Consequently, common summary statistics (e.g., means, medians, standard deviations) do not adequately describe the data. The following sections discuss the findings in more detail.

### Effect of location

The present study is an illustrative example of the complex interactions that can occur between the physical and social environment in a group.

We speculate that in MA1 movement was still primarily influenced by the initial boundary conditions of the experimental set-up (physical environment). With increasing distance from the waiting area, the influence of the environmental conditions decreases and the effects of the social environment become more influential (MA2-3). By changing the geometry (bottleneck, MA4), the steady state becomes transient again and the influence of the physical environment increases.

### Effect of width

The two bottleneck widths chosen for this study were relatively narrow. Yet, even the small differences between the narrow (0.9 m) and wider (1.2 m) bottleneck caused participants to move slightly faster in the wider bottleneck conditions (around 0.05 meter/s in MA2 and MA3).

The dimensions of the PWD’s assistive devices offer a possible explanation: the average width of the wheelchairs used was ${0.6}{} \pm {0.08} \text {m}$. With the passage widths used, this resulted in a lateral distance to the enclosing walls in the bottleneck of 0.15 m to 0.30 m on both sides. While the additional width was not sufficient to allow passage of an additional person it may have slightly eased the passage for PWDs. To quantify this observation, further studies with larger additional widths (e.g. with a multiple of the wheelchair widths) are necessary.

### Effect of crowd heterogeneity

The most noticeable effect on the parameters is the influence of *Crowd heterogeneity*. While the data on the speed of the PWoD were consistently normally distributed, those of the PWDs were not. In particular, participants in wheelchairs reduced their speed at a significantly greater distance from the bottleneck, even though they already moved at slower speeds.

Low densities (MA1) lead to a wide spread of velocities in the *mix*-condition due to the wide range of assistive devices used (cane for the blind, manually operated wheelchair, electrically operated wheelchair, wheelchair operated by an assistant, ...). In the subsequent MAs (MA2-3) and with increasing proximity to the bottleneck entrance, the distributions widened for both populations, but especially for PWDs. Individual speed in MA2 prepared participants from the *whe* group for passage into the bottleneck. Immediately before the bottleneck (MA3), increased variance in speed was noticeable for PWoD. At these locations, the ordering process for entering the bottleneck was completed, and the PWoD either closed gaps or resumed their movement after waiting (the number of observed behavioural actions also decreased at this location).

Descriptively, the distance between neighbours in homogeneous crowds (*ref*) decreased as participants moved closer to the bottleneck, while the distribution became wider shortly before entering the bottleneck (cf. Fig. 5). The location from which the distance between neighbours increased again after passing the bottleneck depends on the time (and place) at which entry into the bottleneck is arranged by the individuals.

**Figure 8:**
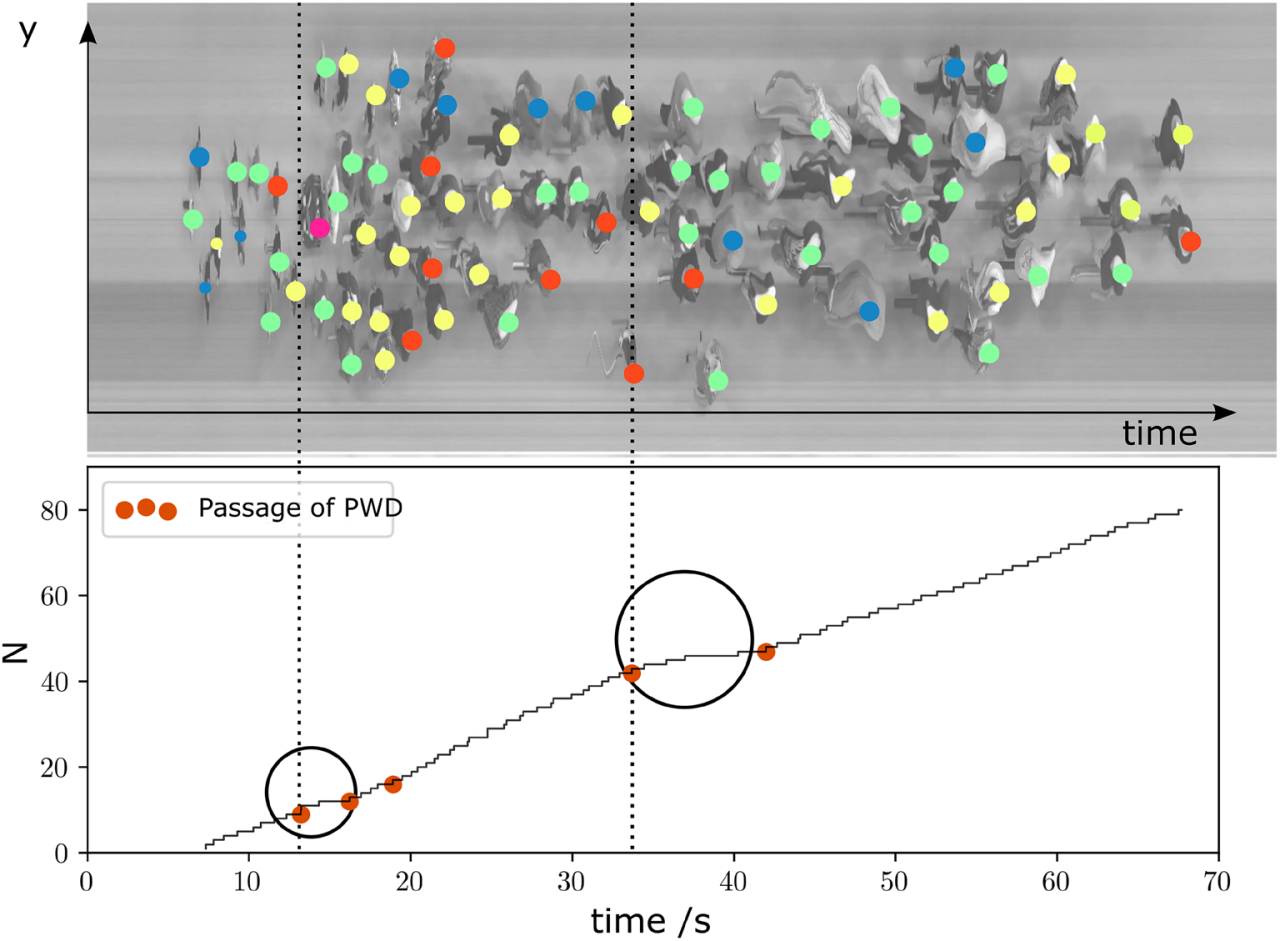
Spatio-temporal representation (top) and passage over time plot (bottom) in a bottleneck study under crowd heterogeneity mixed conditions (passage width of 1.2 m). For the spatio-temporal plot, an image sample is obtained framewise (framerate: 25 s$^{-1}$) along a vertical line at $x=-{2.4} m$ (cf. red vertical line in Fig. 1) with a height of 600px and a width of 1px from the footage. The space along the line in Fig. 1 is represented by the vertical axis, while the horizontal dimension is the time. The crossing of the bottleneck entrance is plotted cumulatively in the bottom plot. The passage of PWD is highlighted by the orange dots (and often recognizable by a subsequent longer interval without a passage).

One potential explanation of this observation are differences in passing behavior across the heterogeneity conditions. Qualitative analysis of the footage showed that wheelchair users were passed by PWoDs only hesitantly from a certain distance to the bottleneck (cf. for example Fig. 8). This hesitation (i.e., slowing down) increased the distance to the preceding participant, especially when the preceding participant was easily identifiable as a PWD. This had the effect that “gaps” opened in front of the PWDs.

Consequently, the distance between neighbours is also dependent on the *crowd heterogeneity* in MA2 and MA3. In the *whe* group, the distance between neighbours was higher than in *ref*. However, the distance between neighbours was lowest in the *mix* group. We speculate that this was mainly due to the influence of the companions for the PWD. In the *mix* group, four PWDs had a personal companion (cf. Supplementary Data 3), who always stayed in proximity to their client.

We observed some interesting patterns regarding ‘jostling’ behavior, which was observed most frequently in the *ref* group. This behavior was observed almost without exception at the end of the crowd and towards the end of a day of data collection. In addition, almost always the same participants were involved in these actions. We speculate that participants motivation and focus waned at the end of a long day of data collection, which may have enticed them to show this behavior. This lends itself to interesting hypotheses with regard to anti-social behavior in crowds. For instance, future research could explore if fatigue increases the likelihood of unsafe behavior in crowds.

In both heterogeneous groups, a higher frequency of behavioural actions was observed, which also depended on the distance to the bottleneck. In particular, one- and two-armed gestures in combination with waiting indicate a higher need for communication and to regulate priority. It emerged from the observational studies that waiting in the crowd was not just a necessity to maintain interpersonal distance from neighbours. Rather, waiting was often an active gesture that signalled priority as an implicit behaviour at a bottleneck or in areas with queues.

Turning, for example, is an ambiguous activity. On the one hand, turning may be the consequence of an overtaking maneuver or to detect the difference in speed between two neighbours. It can also be an expression of turning towards each other in conversation. On the other hand, turning can also be interpreted as a necessary action that widens a person’s field of vision and determines the direction of movement of future steps. Since this activity is no longer necessary after the entrance of the bottleneck, the number of turns slightly increased in MA2 and decreased significantly after passing the entrance of the bottleneck (MA4).

Both, the adaptations in speed and the stability of the neighbourhoods can be further explained with a qualitative observation. Figure 8 shows a spatio-temporal plot^48^ from a video recording ($w={1.2} \text {m}$). An image sample is obtained framewise (framerate: 25 s$^{-1}$) along a vertical line 2.4 m in front of the bottleneck entrance (cf. Fig. 1) with a height of 600px and a width of 1px. In this plot, the space along the line is represented by the vertical axis, while the horizontal dimension is the time. The longer a participant spent at the defined location, the more “stretched” they appear in the top of Fig. 8.

Two participants with specific characteristics of interest are highlighted by circles at the bottom of Fig. 8. Both participants used a clearly visible assistive device—a wheelchair and a white cane. And both were surrounded by PWoDs (these were guides) who adjusted their speed to that of the PWD and did not pass. As a result, unused “time-space-slots” appeared in the neighbourhood of these participants. Later on, the crowd separated into three parts: a group ahead of the wheelchair user moved with at unrestricted speed of PWoDs; around and behind the wheelchair user was a group with participants who adapted to the speed of the wheelchair user; and, at the end, a group emerged that moved slowly and considerately around the participants with the white cane. Once the speeds were adjusted, the neighbourhood distances remained stable.

### Effects on flow

The findings on flow rates at the bottleneck are consistent with findings in the literature showing that increasing bottleneck widths improves flow rates (cf. Fig. 5.20 in Geoerg (2020)^31^). In addition, homogeneous crowds moved with less effort through bottlenecks than heterogeneous crowds. The differences in flow rates and time gaps were largely driven by the presence of PWDs, as can be seen by the data distribution in Fig. 6. Descriptively, there were often more people next to and behind PWDs than would have been observed in a situation with a person without a disability. We argue that it is these spatial and temporal gaps that create heterogeneous conditions in the specific flow (Fig. 8), which can lead to a reduction in overall flow rate for heterogeneous crowds (Table 5).

While these findings underline the importance of studying heterogeneous crowds, the analyses presented above also clearly show that observing average flow rates alone does only paint an incomplete picture. In fact, given that the time gap data were rarely normally distributed (and even bimodal in one case; Fig. 6) indicate that average flow rates do not represent the data well. In some cases, the reliance on average values risks that assumptions about flow rates may be overly optimistic for heterogeneous crowds. Therefore, we argue that future studies of crowd dynamics should go beyond traditional measures such as flow rates in order to better understand human crowd dynamics.

## Limitations and future work

There are several limitations to the current study. Firstly, certainly the most critical limitation is that the densities observed in the present study fall into the free-flow branch of the fundamental diagram, where density is smaller than 3 m$^{-2}$^31^ and does not completely reach the capacity of the geometry. Thus, no predictions can be made whether the increased distances to neighbours with impairments and the resulting larger areas available would also emerge under higher densities. In addition, the current study did not include a simulated emergency scenario. That is, it is unclear whether the results can be generalized to situations in which people have strong motivations to pass through a bottleneck. By controlling a crowds density and individual motivation in future studies, important insights can be gained into the validity of polite behaviour and the influence of interaction on pedestrian dynamics.

Secondly, although the type of visible impairment was varied by the use of different assistive devices in the presented study, the specific mode of use of these assistive devices was not controlled. Thus, neither the required fixed space requirement of the assistive devices nor the mode of locomotion (manually self-or electrically operated, pushed by an assistant) was systematically controlled. The dimensions of the wheelchairs used varied in length of ${1.07}{} \pm {0.19}m$ and in width of ${0.68}{} \pm {0.07} m$. As a result, a wheelchair had a physically based incompressible space requirement of ${0.73}{} \pm {0.15} m^{2}$, which does not take into account the additional space required by any accompanying persons or safety distances. It has not been conclusively clarified whether devices with a fixed space requirement used in everyday life (e.g. luggage, strollers) lead to similar behaviour in the group and whether the space requirement of the assistive device is the cause or whether the observed phenomena result from a disability being visible. If the latter is the case, the reason for the distance to people with assistive devices should be investigated.

Thirdly, the study was conducted under controlled experimental conditions. The strength of this approach is the ability to test specific hypotheses. However, the overall artificial context of the data collection limits the degree to which their findings can be generalized beyond the laboratory. However, we argue that the fact that we observed unexpected and in some cases socially undesirable behavior (e.g., jostling) supports the relatively high degree of ecological validity of the present study. Nonetheless, future studies are needed to explore the link between laboratory and field observations.

Fourthly, the PWDs in the present study were not a homogeneous group; PWDs differed in their disabilities and assistive devices (see Table 3). In addition, PWDs were on average about 10 years older than PWoDs. These aspects limit the experimental control. For example, it is possible that some of our findings could be explained by age differences. Future studies should carefully control these potentially confounding factors, for example by recruiting wheelchair users with similar mobility and age profiles.

Fifthly, behavioral observations of any kind are strongly dependent on the cultural set of values and context. There are not only cultures in which lower (or higher) distances or individual speeds are observed between individuals (e.g.^49–54^). Granting precedence as a gesture of politeness is a result of the individual’s interpretation of the context and his or her social value framework. The behaviors observed here are not generalizable worldwide, but are embedded in the Western European social context. The studies were planned and conducted before the onset of the SARS-Covid 19 pandemic. It is plausible to assume that study participants today, with the experience gained from the pandemic, would find it more difficult to voluntarily accept high densities of people. Nevertheless, studies today are designed with great counter-measure efforts to counteract the risk of infection. That these efforts will be communicated to participants and that subjects will only agree to participate after an individual cost-benefit consideration, we assume that the observations presented here can be replicated with sufficient time lag from the pandemic. Sixthly, note that the present work explores individual interactions between pedestrians, but does not explicitly consider the formation of stable groups. We occasionally observed group like behavior (e.g., participants walking next to the same person repeatedly, jostling people they know). Spontaneous group behaviors certainly may have influenced the outcome of the present study. Future work should either systematically explore these effects or try to minimize its occurrence (e.g., by randomizing start positions of participants). However, a more formal analysis is beyond the scope of the present paper but should be considered in future work. The interested reader is also pointed to relevant work on group behavior within the context of pedestrian dynamics (e.g.,^55–60^).

Finally, the behavioural analysis was conducted ex-post. We have no internal insights into the motivation and thoughts of the participants. It remains an open and important question what visible and invisible characteristics of the neighbouring persons motivate someone to maintain the distance. For instance, future studies should investigate which of the phenomena observed in the present study are influenced by social norms.

## Conclusions

The presented study provided insights on some complexities in crowd dynamics that emerge in heterogeneous crowds. Using trajectory data from wheelchair users, pedestrians with otherwise reduced mobility, as well as participants without disabilities, we analyzed interpersonal distances between neighbours, the adaption of speed and the behavioural activity. These microscopic quantities of crowd dynamics then gave rise to macroscopic differences in flow rates in the present study. Most notably, the presence of participants with disabilities influenced the behaviour of their neighbours in the crowd. For instance, the speed of the neighbours was adapted to the speed of the PWD, passing was observed less frequently, and the interactions between participants to self-organize movement in front of the bottleneck increased.These effects did not only affect summary statistics such as the average flow rates, but also the overall distribution of the data.

Taken together, these findings question the validity of the standard calculations of density-dependent flows for safety-related parameters in engineering tools that are commonly used for planning purposes. For instance, the analysis has shown that for the studied heterogeneous populations, increased space requirements are to be expected due to the increased interactions between people. Clearly, further research is needed to understand the dynamics of realistic, that is heterogeneous, crowds.

## Supplementary Information

Supplementary Information.

## Data Availability

The data that support the plots and findings of this paper are openly and long-term available in the ‘Pedestrian Dynamics Data Archive’ hosted by the Institute of Civil Safety Research (https://ped.fz-juelich.de/da/doku.php). To access the data, proceed to the following DOI: https://doi.org/10.34735/ped.2017.1.
