# Supplementary material for: How people with disabilities influence crowd dynamics of pedestrian movement through bottlenecks
Source: Sci Rep. 2022 Aug 22;12:14273. doi: 10.1038/s41598-022-18142-7 (PMC9395390; doi:10.1038/s41598-022-18142-7)
Supplement: Supplementary file 1 — Supplementary Information. [file 41598_2022_18142_MOESM1_ESM.pdf]

Supplementary information

Supplementary information 1: Behavioral actions coding manual

The frequencies of the behavioural actions were determined using objectified visual video transcription. For this purpose, observable events (behavioural actions) and visually recognisable population memberships of participants (overtly visible disabilities) were documented in a temporal and spatial context. Observations were operationalised in the coding manual presented below.

Supplementary Table T1: Coding manual.

Category	Subcategory	Description
Turn over		A rotation of the head and/or upper body by 30° without the orientation of the whole body deviating from the main direction of movement.
Gesture	single-armed	A gesture[a] made with one arm or hand and addressed to another person.
	double-armed	A gesture[a] made with both arms or hands and addressed to another person.
Overtly visible disabled person	Accompanying person	A person visibly accompanied by a second person (but not wheelchair users).
	Technical assistance	Person identifiable by a technical assistive device (e.g. walking stick, white stick...), but not a wheelchair.
	Wheelchair (self-operated)	Person sitting in a manually driven wheelchair (and moving it without external assistance).
	Wheelchair (electrically powered)	Person seated in (and independently controlling) an electrically powered wheelchair.
	Wheelchair (pushed by an attendant)	Person seated in a wheelchair (manually driven or electric) whose locomotion/control is mainly realised by a second person. The person in the wheelchair and the assistant are regarded as a unit.
	Other	Overtly identifiable as an disabled person by other characteristics.
Misc	Wait	Person who does not change position for at least 1 s.
	Jostle	Participants did not follow instructions consistently, for example, we observed that some participants occasionally jostled each other playfully.

Category	Subcategory	Description
	Touching walls	Touching, groping or tapping the walls.

[a] Excluded are: Leaning on another person or wall, brushing through the hair, holding the hand in front of the mouth while coughing, putting the hands in the pockets of the trousers and crossing the arms in front of the chest.

Supplementary information 2: Coding manual for visible attributes of participants

An important feature of the presented studies is the consideration of individual mobility characteristics (operationalised by disabilities). At the same time, assumptions were made to cluster the individual characteristics and to assign generic terms. But the level of experience and familiarity of the participants without disabilities in dealing with people with disabilities is not known. Nevertheless, the presence of persons with visible disabilities (i.e., a person who lives with an overtly visible disability such as a wheelchair user) is recognisable to an observer and can influence the movement of the group and behaviour. In order to make the influence of the presence of PWD on the behaviour of all participants in an observed study situation interpretable, a standardised narrative description of participants with disabilities based on a phrasing (Tab. T2) template was developed.

Supplementary Table T2: Coding manual for the narrative description of PWD.

Main category	Attribution	Categorisation	Specification
Gender	male ∨ female ∨ not specified	–	–
Assistive device	Personal ∨ Walking frame ∨ Walking stick ∨ White cane ∨ White stick ∨ Wheelchair	electrically operated ∨ manually operated	by hand ∨ by chin
Body region	arm ∨ body ∨ brain ∨ hands ∨ head ∨ foot ∨ knee ∨ leg ∨ palms ∨ shoulder	–	–
Adjective	hang down ∨ gesticulation ∨ knock-knees ∨ obese ∨ often stretched out ∨ playing ∨ saliva flow ∨ spasticity ∨ sporty ∨ is swinging forth and back ∨ talkative ∨ tilts	wildly ∨ strong ∨ unilateral ∨ bilateral ∨ to the side	–

Main category	Attribution	Categorisation	Specification
Features	assistance ∨ distanced to ∨ disturbed balance ∨ following ∨ gait posture ∨ gait style ∨ hands ∨ look around ∨ nervous ∨ operating ∨ orientation difficulties ∨ oriented to ∨ holding the shoulder of a predecessor ∨ outgoing ∨ reaction ∨ seeking for information ∨ self-confident ∨ step frequency ∨ step size ∨ strained ∨ turned to the outside (hands)	personal ∨ others ∨ environment ∨ neighbours ∨ person of trust ∨ cool ∨ upright ∨ respectfully ∨ distanced ∨ angled ∨ springing ∨ agile ∨ jerky ∨ maneuverable ∨ swaying ∨ bent / bended ∨ uses foot to push off ∨ limps ∨ wiggles ∨ see sawing ∨ on body ∨ spread out ∨ frequently ∨ once in a while ∨ single-handed ∨ two-handed ∨ neighbours ∨ person of trust ∨ environment ∨ all directions ∨ delayed ∨ fast ∨ fast ∨ slow ∨ influenced by shortened legs ∨ small ∨ large	wildly ∨ fixed ∨ various ∨
Characteristics	activity ∨ appearance ∨ articulation ∨ communication style is ∨ curious ∨ deflect ∨ distancing ∨ impairment ∨ interested in ∨ may stand for a short time ∨ no characteristic features ∨ requires assistance for ∨ self-initiative ∨ slightly annoyed ∨ sociable behaviour	high urge ∨ insecure ∨ introverted ∨ friendly / polite ∨ open-mindedness ∨ self-confident / cool ∨ tired ∨ difficulties ∨ hard to understand ∨ not possible ∨ slowly ∨ silent ∨ communicative ∨ by images ∨ only with trusted persons ∨ easy to ∨ undistanced ∨ distanced ∨ seeing ∨ hearing ∨ walking ∨ mental ∨ seeking attention from others ∨ environment ∨ other persons ∨ short distances ∨ long distances ∨ hardly any ∨	–

The result is a description of persons who, when viewed from an observation view, are

visible (and recognisable) and objectifiable as having a disability. The description is based on a five-step process:

1. Identifying behavioural phenomena,
2. Coded description of the observation,
3. Revision of the descriptions and clustering (coding manual),
4. Encoding footage by using the coding manual,
5. Supplement and review coded keywords by a second rater.

For further details, we refer to Georg (2021) [1].

PWD_34: A *female* participant who *is* assisted by a *manually operated wheelchair* (l_{whe} = 1.40 m, w_{whe} = 0.6 m) *with personnel assistance.* Participant's *body* is *sitting in a wheelchair.* The individual motion style is characterised by *dependency to be pushed, orientation towards a contact person.* A third person *will* notice *articulation is not possible due to bilateral spasticity* in everyday life situations.

PWD_35: A *male* participant who *is* assisted by an *manually operated wheelchair* (l_{whe} = 1.16 m, w_{whe} = 0.74 m) *and the body often tilts to the side.* Participant's *body* is *sitting in a wheelchair.* The individual motion style is characterised by *slow operating single-handed.* A third person *will* notice *the unilateral spasticity and interested behaviour in the environment* in everyday life situations.

PWD_36: A *female* participant who *is* assisted by an *electrical operated wheelchair* (l_{whe} = 1.23 m, w_{whe} = 0.64 m) *with chin steering.* Participant's *body* is *sitting in a wheelchair.* The individual motion style is characterised by *slow movement.* A third person *will* notice *articulation difficulties and hard to understandable communication* in everyday life situations.

PWD_37: A *female* participant who *is* assisted by an *electrical operated wheelchair* (l_{whe} = 1.10 m, w_{whe} = 0.77 m) *with (sporadic) personnel assistance.* Participant's *body* is *obese and sitting in a wheelchair.* The individual motion style is characterised by *jerking by operating with arms and feet and orientation towards environment.* A third person *will* notice *an open-mindedness appearance* in everyday life situations.

PWD_38: A *female* participant who *is* assisted by an *manually operated wheelchair* (l_{whe} = 0.92 m, w_{whe} = 0.66 m) *with personnel assistance.* Participant's *body* is *sitting in a wheelchair and the arms are very short.* The individual motion style is characterised by *fast and maneuverable.* A third person *may* notice *no characteristic feature* in everyday life situations.

PWD_39: A *female* participant who *is* assisted by an *manually operated wheelchair* (l_{whe} = 0.90 m, w_{whe} = 0.60 m) *with personnel assistance.* Participant's *body* is *sitting in a wheelchair.* The individual motion style is characterised by *no specific feature.* A third person *may* notice *no characteristic feature* in everyday life situations.

PWD_40: A *male* participant who *is* assisted by an *manually operated wheelchair ($l_{whe} = 0.76$ m, $w_{whe} = 0.66$ m).* Participant's *body* is *sitting in a wheelchair.* The individual motion style is characterised by *fast and agile; uses foot to push off and* . A third person *may* notice *no characteristic feature* in everyday life situations.

PWD_48: A *female* participant who *is not* assisted by an *assistive device.* Participant's *body* is *characterised by strong limitation of vision.* The individual motion style is characterised by *a slightly bent gait posture and high step frequency.* A third person *may* notice *a good orientation* in everyday life situations.

PWD_49: A *female* participant who *is not* assisted by an *assistive device.* Participant's *body* is *characterised by severe hearing impairment.* The individual motion style is characterised by *orientation to neighbours and hardly any self-initiative.* A third person *may* notice *no characteristic features* in everyday life situations.

PWD_51: A *male* participant who *is* assisted by a *manually operated wheelchair ($l_{whe} = 1.10$ m, $w_{whe} = 0.80$ m) and personnel assistant.* Participant's *body* is *characterised by sitting and fixed to a wheelchair.* The individual motion style is characterised by *no characteristic feature.* A third person *may* notice *an open-mindedness appearance* in everyday life situations.

PWD_52: A *female* participant who *is* assisted by a *personnel assistant.* Participant's *body* is *heavily bent.* The individual motion style is characterised by *difficulties in orientation and dependence on hand-guidance by others.* A third person *may* notice *no characteristic features* in everyday life situations.

PWD_54: A *female* participant who *is* assisted by a *white stick and personnel assistant.* Participant's *body* is *characterised by no characteristic features.* The individual motion style is characterised by *orientation by hand and usage of arms for orientation.* A third person *may* notice *no characteristic features* in everyday life situations.

Supplementary information 3: Speed density relations by crowd heterogeneity

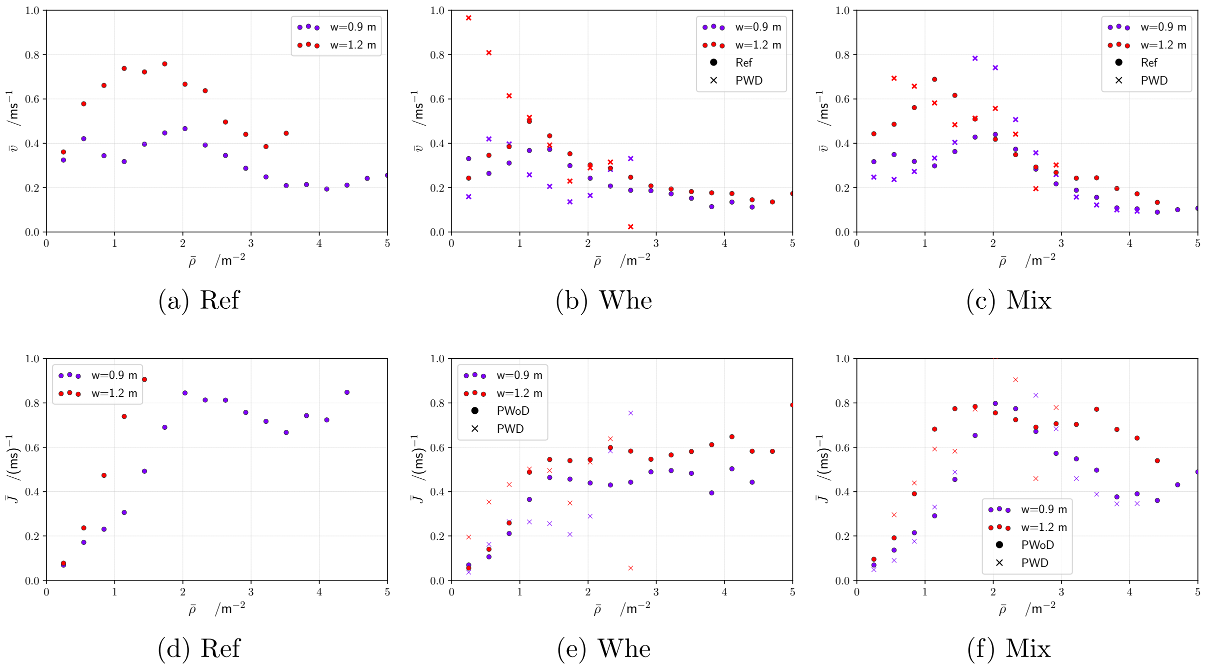

Supplementary Figure S1: Empirical relations $\bar{v}(\bar{\rho})$ (a)-(c) and $J_s(\bar{\rho}) = \bar{v} \cdot \bar{\rho}$ (d)-(f) according to the Crowd Heterogeneity.

Supplementary information 4: Condition of participation and consent

Participation in the movement studies is only possible for persons of legal age after confirmed registration. Participation is voluntary. Registration on the day of the movement studies will only take place upon presentation of an identity card. According to the confirmation of registration, participation will take place as a participant or as a helper.

The movement studies will start on Saturday, June 10, 2017 and Sunday, June 11, 2017 at 9 am and end at approximately 5 pm. Registration for participation can be for one or both days. The registration requires a punctual appearance. Those who do not show up on the first day despite registration will not be allowed to participate on the second day.

Each participant is obliged to follow the instructions of the organizers and their representatives. There is a legal accident insurance for any injuries that the participants and the helpers may suffer during the movement studies.

During the movement studies, photographs, audio and video recordings will be made. Those who participate in the movement studies thereby declare their consent that their image and audio data may be used for scientific purposes, publications and media reporting.

For participation in the movement studies, the test participants and helpers will be compensated with a50 euros per day (or 25 euros if only participating in half a day). If the event is cancelled, there is no right to payment of the honorarium.

The participant or the helper is responsible for taxation. Travel expenses etc. will not be reimbursed. The fee will be paid in cash. The payment requires that the participant or the helper has participated in the movement studies for a full or half day.

IMPORTANT: Each subject will receive a personal colored cap upon enrollment. This cap may not be exchanged or passed on with other test persons during the day. For technical reasons, we ask that you do not wear light-colored clothing.

The organizer is not liable for valuables and the participants' wardrobe.

References

[1] Paul Geoerg. *The Influence of Individual Characteristics on Crowd Dynamics: Phd thesis.* Jülich: Forschungszentrum Jülich GmbH, Zentralbibliothek, Verlag, 2021. ISBN: 978-3-95806-561-1.
